# Factors affecting forest area change in Southeast Asia during 1980-2010

**DOI:** 10.1371/journal.pone.0197391

**Published:** 2018-05-15

**Authors:** Nobuo Imai, Takuya Furukawa, Riyou Tsujino, Shumpei Kitamura, Takakazu Yumoto

**Affiliations:** 1 Department of Forest Science, Tokyo University of Agriculture, Setagaya, Tokyo, Japan; 2 Center for Biodiversity, Forestry and Forest Products Research Institute, Tsukuba, Ibaraki, Japan; 3 Center for Natural Environment Education, Nara University of Education, Takabatake-cho, Nara, Japan; 4 Department of Environmental Science, Ishikawa Prefectural University, Nonoichi, Ishikawa, Japan; 5 Primate Research Institute, Kyoto University, Inuyama, Aichi, Japan; University of Maryland at College Park, UNITED STATES

## Abstract

While many tropical countries are experiencing rapid deforestation, some have experienced forest transition (FT) from net deforestation to net reforestation. Numerous studies have identified causative factors of FT, among which forest scarcity has been considered as a prerequisite for FT. In fact, in SE Asia, the Philippines, Thailand and Viet Nam, which experienced FT since 1990, exhibited a lower remaining forest area (30±8%) than the other five countries (68±6%, Cambodia, Indonesia, Laos, Malaysia, and Myanmar) where forest loss continues. In this study, we examined 1) the factors associated with forest scarcity, 2) the proximate and/or underlying factors that have driven forest area change, and 3) whether causative factors changed across FT phases (from deforestation to net forest gain) during 1980–2010 in the eight SE Asian countries. We used production of wood, food, and export-oriented food commodities as proximate causes and demographic, social, economic and environmental factors, as well as land-use efficiency, and wood and food trade as underlying causes that affect forest area change. Remaining forest area in 1990 was negatively correlated with population density and potential land area of lowland forests, while positively correlated with per capita wood production. This implies that countries rich in accessible and productive forests, and higher population pressures are the ones that have experienced forest scarcity, and eventually FT. Food production and agricultural input were negatively and positively correlated, respectively, with forest area change during 1980–2009. This indicates that more food production drives deforestation, but higher efficiency of agriculture is correlated with forest gain. We also found a U-shaped response of forest area change to social openness, suggesting that forest gain can be achieved in both open and closed countries, but deforestation might be accelerated in countries undergoing societal transition. These results indicate the importance of environmental, agricultural and social variables on forest area dynamics, and have important implications for predicting future tropical forest change.

## Introduction

Alteration of land use is one of the major causes of global environmental change which is driving species to extinction and emitting increasing amount of green-house gases. In particular, global deforestation rate is still alarmingly high[1], and the tropics are the only biome to exhibit an increasing trend of forest cover loss in the 21^st^-century[2]. Deforestation and forest degradation in the tropics are responsible for 7–14% of anthropogenic carbon emissions[3] and pose one of the greatest threats to global biodiversity[4]. Therefore, reducing tropical deforestation and even reversing the trend to net forest gain are top priorities of global environmental policy.

While many tropical countries are experiencing ongoing deforestation, some have gone through a transition from net deforestation to net reforestation, as known as “forest transition (FT)”[5]. The FT hypothesis explains forest recovery as a result of abandonment of marginal agricultural land followed by forest regeneration, as well as tree plantation[6][7][8]. Economic development is almost a prerequisite of FT[9][10][11][12][13][14][15], but different pathways have been suggested on how it affects forest recovery. The wealth brought by economic development would enable tropical countries to be financially comfortable enough to invest in reforestation schemes[16] or import wood and food products from other countries while preserving its own forest[13][14][17][18] [19][20]. Economic development may also change the demographic pattern of a country (decrease in rural population with the increase in urban population) through the increase in off-farm employment, which leads to cropland abandonment[5]. Improvement in agricultural productivity is also suggested to encourage abandonment of marginal croplands[21]. Although it may not be a direct result of economic development, democratic societies[22][23][24] or countries with better governance[15][25][26] are suggested to show less deforestation and/or more forest recovery. Despite the diversity of socio-economic factors that have been suggested to be related to FT, most studies have employed a limited number of factors in their analysis. Additionally, various environmental conditions, such as precipitation, temperature, vegetation, and topography, are known to affect forest area change at the local to subnational scales[27][28][29], but their effects have rarely been incorporated in national-scale studies.

**Fig 1:**
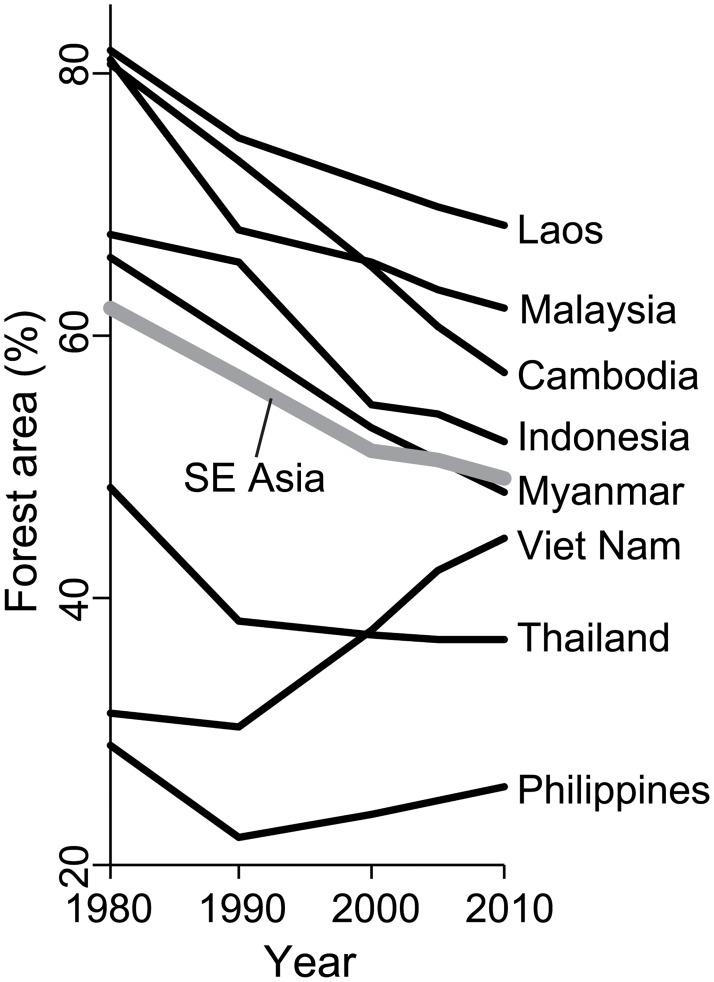
Changes in percentage forest area of the eight SE Asian countries during 1980–2010.

Exhaustion of forest resources is also considered as a prerequisite of a country to experience FT. When forests become scarce, the need for forest conservation is realized with rising price of forest products, or forest protection is promoted in order to restore the deteriorated forest ecosystem services[30][31][32]. Rudel et al. (2005)[31] pointed out that this “forest scarcity pathway” could be more prominent in densely populated Asian countries, compared to less populous Latin American countries. In southeast Asia, forest area stopped to decline in Thailand and increased in the Philippines and Viet Nam since 1990, but the other five SE Asian countries experienced forest loss during 1980–2010 (Fig 1)[1][33]. The three FT countries (Philippines, Thailand and Viet Nam) exhibited lower remaining forest area (30±8%, mean±SD) compared to the other five SE Asian countries (68±6%, Cambodia, Indonesia, Laos, Malaysia, and Myanmar) as of 1990 (Fig 1). This implies that forest scarcity *per se* may have led to FT in the three countries. Although the pattern and processes of FT in the three countries have been well studied[6][14] [34][35][36], clarifying why the three particular countries, but not the other five countries, have already exhausted their forest resources and experienced FT would lead to a better understanding of the entering point of the forest scarcity pathway.

Grainger[7] suggested that, during the FT process, mechanisms underlying the deforestation phase and the subsequent reforestation phase are not identical. However, recent studies reported that factors associated with forest area change are consistent during both deforestation and reforestation phases, while relative importance of each factor varied among phases[15][37]. This implies that there might be a common mechanism across the FT phases, in which a socio-economic factor might initially accelerate deforestation, but then encourage reforestation. Such process could be a key to not only reduce deforestation but also enhance forest recovery.

SE Asia used to experience the fastest rate of deforestation among the tropics especially until the 1990s[38]. Smallholders supported by recolonization programs by the state were considered the main driver of deforestation up to the 1980s, but their role was replaced by private enterprise agriculture until the 1990s[39]. Deforestation continued during the 1990s and 2000s in the region but with a slower rate because of reversing trends in forest area in Thailand, the Philippines, and Viet Nam (Fig 1)[1]. Displacement of deforestation to other countries through timber imports played a big role to achieve forest recovery in Viet Nam[34][35]. Expansion of oil-palm plantation has been one of the major causes of deforestation in Indonesia and Malaysia during this period[39][40]. In Myanmar, commercial agricultural concession, timber extraction and infrastructure development, underlain by international investment, civil war and weak land tenure, were identified as the major drivers of deforestation[41].

To elucidate the general process of FT in SE Asia, we employed 33 socio-economic factors pertaining to proximate (production of wood, food, wood and food aggregated, and export-oriented food commodities) and underlying causes (demographic, social, economic and environmental factors, as well as land-use efficiency, and wood and food trade) of deforestation in eight SE Asian countries at the national scale during 1980–2010. We also examined the relationship between percentage forest area and these causative factors in 1990 to understand the conditions leading to forest scarcity. We addressed three specific questions; 1) what are the socio-economic conditions that lead a country to enter the forest scarcity pathway, 2) which proximate and/or underlying factors have the most significant impacts on forest area change, and 3) whether the relationship with the identified causative factors change across the FT phases (from deforestation to net forest gain)?

## Methods

### Data collection

This study covered eight southeast Asian countries, namely, Cambodia, Indonesia, Laos, Malaysia, Myanmar, the Philippines, Thailand and Viet Nam, encompassing 3 decades (1980–2009) divided into four periods (i.e., 1980–1989, 1990–1999, 2000–2004 and 2005–2009). All countries were analyzed together to extract common mechanisms underlying the entering point of the forest scarcity pathway and the process across the FT phases. Data on remaining forest area (%) and the rate of change in forest area (% yr^-1^) were obtained from the Global Forest Resources Assessment (FRA): data on the 1980s were from FRA 1990[33] and data on the 1990s and onward were from FRA 2010[1]. FRA’s forest area data have been criticized for being variable in quality across countries and for inconsistencies in definitions[42], but it remains the sole comprehensive source of national deforestation rates prior to 2000 (see Hansen et al. 2013)[2]).

We used data on wood and food production as the proximate causes of forest area change. Instead of using production volume, we converted the values into per capita land area required to produce the products (km^2^ person^-1^ yr^-1^) in order to account for the difference in land use impacts (i.e., difference in land area required to produce the same volume of different products). Details of the calculation process is provided in Kastner et al. (2014)[43] and the Supporting Information (S1 Text). This calculation enabled us to directly compare and aggregate the production impact of different wood and food products under the same unit. Wood production covered industrial roundwood including derived products. Food production encompassed almost 450 crops and livestock products, including ten major crops and two groups of commodity crops of interest, namely, oil palm and stimulants (coffee and cocoa).

As for the underlying driving forces of forest area change, we considered demographic, economic, social, and environmental variables, as well as land-use efficiency, and wood and food trade. Demographic variables included population density (person km^-2^), rural, urban and total annual population growth rates (% yr^-1^), and percentage of urban population (Panels a-d in S7 Fig). Economic variables included GDP per capita (PPP adjusted, current international USD), GDP growth rate (% yr^-1^), level of industrialization represented by the share of manufacturing industry (% of GDP), headcount poverty ratio at 1.9 USD per day (% of population), forest rents (% of GDP), total natural resources rents (% of GDP), proportion of forest rents to total natural resources rents (%), and the Human Development Index (HDI, unitless) (Panels e-k in S7 Fig). Social variables included corruption and social openness (Panels l and m in S2 Fig). The Corruption Perception Index (CPI) provided by Transparency International (http://www.transparency.org/research/cpi) was used to represent corruption. Indices of polity and freedom, obtained from Polity IV regime authority characteristics and transitions datasets, INSCR (http://www.systemicpeace.org/inscrdata.html) and Freedom in the world, Freedom House (https://freedomhouse.org/), respectively, were summarized based on principal component analysis (PCA), and the score of its first axis was used to represent social openness. Land-use efficiency included the index of agricultural input (unitless), cereal yield (Hg ha^-1^), and the index of agricultural yield (unitless) (Panels n-p in S2 Fig). Agricultural input was represented by the first axis of PCA among agricultural machines import, pesticides import and fertilizers consumption per unit agricultural area. Similarly, the yield values of major crops were summarized by PCA to represent agricultural yield. The self-sufficiency ratios (SSR, unitless) for wood, food, and wood and food aggregated were used as the indices of wood and food trade. The SSR was defined as:

SSR=production/(production+imports-exports)×100

The SSR was calculated based on land area required for wood and food production (S3 Fig), and area associated with import/export of wood and food in the eight countries (S4 and S5 Figs). Data on import/export values of food were obtained from Kastner et al. (2014)[43], while those of wood were calculated based on various data sources (see S1 Text). Environmental variables included remaining forest area at the beginning of each period (%), median elevation (m), total land area (km^2^), and percentage land area of lowland tropical forests as potential natural vegetation (%). Characteristics of climate and soil summarized based on PCA analyses (S1 Text) were also used in the analyses.

All variables used in the analyses are listed in Table 1. The data sources and details of the calculation processes are described in S1 Text.

**Table 1:** Variables used in correlation analysis.

	Variable	Unit
*Deforestation*		
Remaining forest area	1. Remaining forest area in 1990	%
Forest-area change	2. Forest-area change during 1980–89	% yr^-1^
	3. Forest-area change during 1990–99	% yr^-1^
	4. Forest-area change during 2000–04	% yr^-1^
	5. Forest-area change during 2005–09	% yr^-1^
*Proximate causes*	1. Per capita area required for wood & food production	km^2^ person^-1^ yr^-1^
Wood extraction	2. Per capita area required for wood production	km^2^ person^-1^ yr^-1^
Agricultural expansion	3. Per capita area required for food production	km^2^ person^-1^ yr^-1^
	4. Per capita area required for oil palm production	km^2^ person^-1^ yr^-1^
	5. Per capita area required for stimulants production	km^2^ person^-1^ yr^-1^
	6. Per capita area required for production of ten major crops	km^2^ person^-1^ yr^-1^
*Underlying causes*		
Population	1. Population density	no. km^-2^
	2. Total annual population growth	% yr^-1^
	3. Rural annual population growth	% yr^-1^
	4. Urban annual population growth	% yr^-1^
	5. Percentage of urban population	%
Economy	6. PPP adjusted per capita GDP	Current international dollar
	7. Annual GDP growth	% yr^-1^
	8. Industry, value added (% of GDP)	%
	9. Headcount poverty ratio at $2/day (% of population)	%
	10. Forest rents (% of GDP)	%
	11. Total natural resources rents (% of GDP)	%
	12. Proportion of forest rents to total natural resources rents	%
	13. Human development index	Unitless
Social condition	14. Corruption	Unitless
	15. Social openness (PCA 1 between the following two variables)	Unitless
	Polity	
	Freedom (political right and civil liberty)	
Land-use efficiency	16. Agricultural input (PCA 1 between the following three variables)	Unitless
	Agricultural machines import per unit agricultural area	
	Pesticides import per unit agricultural area	
	Fertilizers consumption per unit agricultural area	
	17. Cereal yield	Mg ha^-1^
	18. Agricultural yield (PCA 1 between yield values of six crops aggregated)	Unitless
Wood & food trade	19. Self-sufficiency ratio in terms of wood products	Unitless
	20. Self-sufficiency ratio in terms of food	Unitless
	21. Self-sufficiency ratio in terms of wood and food	Unitless
Environmental condition	22. Remaining forest area	%
	23. Median elevation	m
	24. Total land area	km^2^
	25. Climatic seasonality (PCA 1 between 12 soil variables)	Unitless
	26. Soil moisture and CEC (PCA 1 between 19 climate variables)	Unitless
	27. Lowland tropical forests as potential natural vegetation (% land area)	%

### Statistical analyses

For all the 33 variables of proximate and underlying causes (Table 1), we calculated the mean values in each of the four periods (i.e., 1980–1989, 1990–1999, 2000–2004 and 2005–2009). We first examined the relationships between percentage forest area in 1990, when forest area in Philippines and Viet Nam began to increase (Fig 1), and the remaining 32 variables in the 1980s by Pearson’s correlation analysis.

We then examined the relationships between the rate of change in forest area (% yr^-1^) and the 33 variables. As a result, 10 out of 33 variables had a significant correlation with forest area change in at least one of the four periods (S7 Fig and S3 Table; see Results). To further analyze the strength of each of the 10 causative factors on the rate of change in forest area during the four periods, we examined the explanatory power of major variables based on multiple regression analyses. We considered wood and food production individually instead of their aggregated values, and excluded headcount poverty ratio since it was not available for Myanmar. We also considered squared terms for variables that changed their correlation coefficient between positive and negative over time expecting that the variables might have altered their relationship with forest area during FT. The multi-collinearity of explanatory variables was examined based on variance inflation factor (VIF). Variables having VIF ≥ 10 were dropped (with preferential omission of squared terms) to avoid severe multi-collinearity[44], leaving a total of 8 explanatory variables (of which only one was a squared term). The full model with all explanatory variables was defined as:

$$\Delta {FA}_{i}\sim \beta_{1}+\beta_{2}{FP}_{i}+\beta_{3}{WP}_{i}+\beta_{4}{POP}_{i}+\beta_{5}{URB}_{i}+\beta_{6}{SOP}_{i}+\beta_{7}{SOP}_{i}^{2}+\beta_{8}{AGI}_{i}+\beta_{9}{WSSR}_{i}+\varepsilon_{i}$$

where Δ*FA*_*i*_ is the rate of change in forest area, *FP*_*i*_ is per capita area required for food production, *WP*_*i*_ per capita area required for wood production, *POP*_*i*_ is population density, *URB*_*i*_ is proportion of urban population, *SOP*_*i*_ is social openness, *AGI*_*i*_ is agricultural input, and *WSSR*_*i*_ is wood SSR. *β*_1~9_ represent model coefficients (i.e., intercept and slopes), *ε*_*i*_ is the error term, and _*i*_ depicts data from each country and time period. Model selection was based on Akaike information criterion for small sample sizes (AICc)[45]. For each candidate model, we calculated AICc weight (which value adds to 1) representing the normalized likelihood of a model in the set of candidate models[46]. The relative importance of variables (IOV; values ranging from 0 to 1) was calculated by adding the AICc weights of the models in which a variable was selected [46]. All statistical analyses were performed using R[47].

## Results

### Remaining forest area

**Fig 2:**
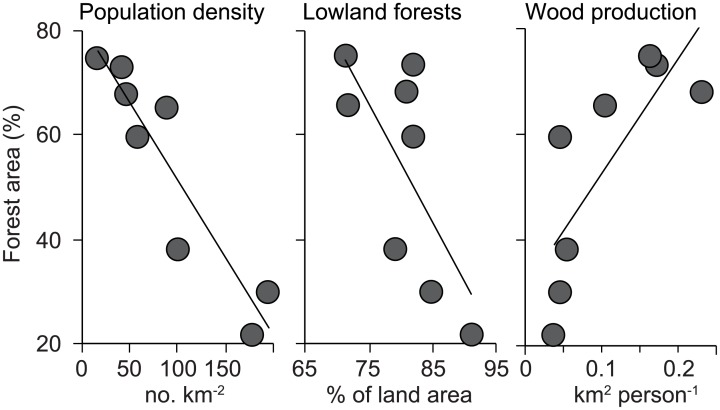
Relationship between remaining forest area as of 1990 and the three variables in which significant correlation at *P* < 0.05 was observed.

Only three out of 32 variables were significantly correlated with remaining forest area in 1990 (*P* < 0.05 for all; Fig 2 and S3 Table). Remaining forest area in 1990 was negatively correlated with population density and potential land area of lowland tropical forests, while positively correlated with per capita area required for wood production.

### Forest area change

As noted above, 10 out of 33 variables showed significant correlations with forest area change in at least once in the four periods (S7 Fig and S3 Table). Proximate causes, including per capita areas required for wood, food, and their aggregated production, were almost negatively correlated with forest area change irrespective of time period (Panel a in S7 Fig). Forest loss was more active in countries characterized by higher social openness and urbanization, and lower poverty ratio in the 1980s, while in countries with lower agricultural input, population density, and higher wood SSR and remaining forest area after the 1990s (Panel b in S7 Fig).

**Fig 3:**
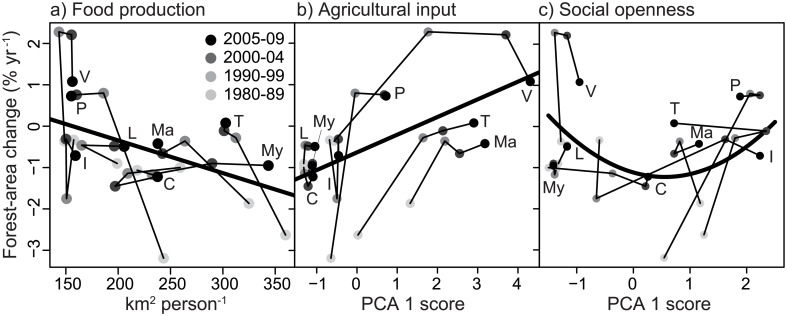
Relationship between rates of forest-area change and per capita areas required for food production (a), the index of agricultural input (b) and social openness (c). Regression lines (bold) are the best-fit multiple regression model from S4 Table. Each country (C, Cambodia; I, Indonesia; L, Laos; Ma, Malaysia; My, Myanmar; P, Philippines; T, Thailand; and V, Viet Nam) is connected by thin lines following the time periods.

Among these candidate variables, model selection of the multiple regression analysis suggested that forest area change during 1980–2009 was best explained by per capita areas required for food production, agricultural input, and both the normal and squared terms of social openness (S4 Table). These four variables had high IOV values compared to the other explanatory variables. The rate of change in forest area decreased with increasing food production (Panel a in Fig 3), while it increased with increasing agricultural input (Panel b in Fig 3). Since the regression coefficient of the squared term of social openness was positive, the relationship between social openness and forest-area change was predicted to be U-shaped (Panel c in Fig 3). In other words, forest area rapidly decreased when the society was moderately open, while forest area increased when the society was either very open or closed.

## Discussion

Demographic and environmental factors were major causative factors showing correlation with forest scarcity in the SE Asian FT countries. In 1990, when the three FT countries were ending their deforestation phases, remaining forest area was negatively correlated with population density and potential area of lowland tropical forests (Fig 2). The fact that high population pressure leads to a low remaining forest area is consistent with many previous studies (e.g., Li et al. 2015[14]). Flatter topography and warmer climate in lowland tropical forests result in a greater accessibility and biomass productivity while a lower cost of clearing forests and transporting products to markets, relative to montane forests. Such lands are susceptible to land-use conversion from forests to cropland[29]. The three FT countries with little forests were the lowest 3 countries for per capita wood production in the 1980s (Fig 2), suggesting that less forest area has concomitantly induced the scarcity of forest resources. Thus, abundance of accessible and productive forests and high population pressures might have contributed to the scarcity of both forests and their products in the three FT countries as of 1990.

Crop production has been a major cause of deforestation in SE Asia in the past three decades, as shown by our multiple regression analysis (Panel a in Fig 3 and S4 Table). Wood production, on the other hand, had minor influence on the overall change in forest area. However, one should note that wood harvest can alter a vast area of forest cover (not forest land use)[17] which can bring other ecological impacts[48]. Smaller or decreasing per capita food production may directly result in reducing deforestation or even net reforestation. Agricultural input and yield have been suggested to have two contrasting impacts on forest area[49]. The first pathway predicts reduction in deforestation or enhancement of reforestation through declining demand for new agricultural land and increasing abandonment of low-yield agricultural land due to higher costs for agricultural inputs and increase in off-farm employment[50]. The second pathway predicts further conversion of forest land to agriculture due to higher agricultural profitability[21][49][51][52][53]. Our multiple regression analysis found that forest area increased with increasing agricultural input (Panel b in Fig 3), while agricultural yield did not correlate with forest area change (S3 Table). This partially supports the first pathway, implying that increase in agricultural investments and decrease in total agricultural production were the key for forest transition in SE Asian forests throughout the study period. Most of the best quality cropland is already used for agriculture. Sustainable and high-yield agriculture within such croplands can contribute to avoiding further agricultural expansion on marginal land and increasing abandonment of low-yield cropland[54], although such agriculture requires larger inputs of fertilizers and pesticides that might cause other environmental issues.

Some studies have suggested that positive forest trends are more likely to occur in democratic societies (e.g., Mather et al.[55][56]). Our results suggested that not only open democratic countries but also countries with autocratic governments experienced forest gain (Panel c in Fig 3). Democratic countries usually have social structure, institutions and political processes which directly or indirectly favor forest conservation, such as through environmental protection organization, market forces and private land ownership for effective resource use, independence of press and free election[22]. Stronger governance in laws, currency and monetary policies also encourage overseas-based corporations to invest in forestry schemes[30]. In contrast, closed and autocratic countries with a strong technocratic basis can forcefully and strictly implement environmental actions than open countries [57]. Our results elucidated that countries in societal transition from closed to open societies are the ones experiencing the highest deforestation rates[58], and international support for forest conservation is most needed in these countries (namely, Cambodia and Myanmar).

Although our analysis covered until 2010 due to limitation in explanatory variables, the latest FRA2015[59] reported that deforestation rates are slowing or forest transition may occur in several other SE Asian countries. Laos and Malaysia could be the next to experience national forest transition, but net forest gains might be still too small to make a firm statement given the uncertainty arising from potential errors in statistics (e.g., uncertainty around the turning point itself)[60]. Indonesia’s net forest loss has dropped by two thirds, from 1.9M ha y^-1^ in the 1990s to 0.68 M ha y^-1^ in 2010–2015[60], and a model prediction study based on FRA2015 suggests that Indonesia will achieve forest gain within the next 15 years (2015–2030)[61]. Recent positive trends in Malaysia and Indonesia are in agreement with our results, because the two countries exhibited relatively low remaining forest area and per capita food production, along with higher social openness, agricultural input and yield. The fact that Laos might reach FT is intriguing, because socio-economic and environmental conditions in Laos contrast with those in the countries showing a positive trend in forest area (S2 Fig). Implementation of policy measures related to reducing poverty, unsustainable wood extraction and slash-and-burn cultivation while encouraging tree plantation have been suggested to contribute to forest recovery in Laos[62]. Despite the increasing number of countries showing positive trends in forest area, primary forest area in SE Asia is still declining owing to the ongoing rapid deforestation in Cambodia and Myanmar[59]. Distinguishing different forest types (i.e., primary, secondary, and plantation), incorporating forest degradation as objective variable and the effects of specific policy measures might lead to a more comprehensive understanding of forest resource dynamics in SE Asia.

As globalization of the economy has accelerated since the end of the 1980s, the three FT countries increased wood imports as if complementing the scarcity of forest resources to satisfy the domestic wood demand (S4 Fig and Panel A in S6 Fig). This contrasted with the other five forest-rich countries which increased wood production and exports. Rehabilitation efforts also increased in the three FT countries from the 1990s onward [16], leading to higher proportion of forests as plantation in the three FT countries (17.0±10.6%) than in the other five countries (3.6±3.3%) as of 2010[1]. These phenomena are in agreement with many recent studies[13][14] [18][20][35], reporting that wood and/or food importer countries often show net forest gain through preserving their forest by a displacement of land use to other countries, although wood and food trade showed little effect on forest area change in our analysis. National strategies aimed at increasing wood and food imports, forest protection, and sustainable forest management often have unintended, detrimental impacts on forests abroad resulting from international displacement of land use[63]. A comprehensive global analysis on the effects of displacement, socio-economy, and policy interventions between FT and non-FT countries is crucial for understanding the key conditions and interventions for a positive forest trend at the global level. Environmental factors, such as topography or areas with productive forests, might also be important preconditions to consider in such global analysis as our study suggested.

## Supporting information

S1 Fig Procedures to calculate areas required for wood production (a), and areas associated with exported/imported wood products (b), paper and pulp (c). (PDF)

S2 Fig Changes in demographic (a-d), economic (e-k), social variables (l, m), and land-use efficiency (n-p) of the eight SE Asian countries. (PDF)

S3 Fig Changes in area required for wood and food production in the eight SE Asian countries during 1980–2009. Data on food production are available only in the period 1986–2009.
(PDF)

S4 Fig Changes in area required for wood export (above zero) and import (below zero) for the eight SE Asian countries by wood categories during 1980–2009. (PDF)

S5 Fig Changes in area required for wood and food export (above zero) and import (below zero) for the eight SE Asian countries by categories during 1986–2009. (PDF)

S6 Fig Changes in self-sufficiency ratio (SSR) in terms of wood (a), food (b), wood and food aggregated (c) in the eight SE Asian countries. (PDF)

S7 Fig Correlation coefficient between rate of change in forest area and proximate causes (a) and underlying driving forces (b) in each of the four study periods. Relationships significant at *P* ≤0.1 in at least one of the four periods only are shown. Black, grey, and white bars are relationships with *P*≤ 0.05, 0.05< *P* ≤0.1, and *P*>0.1, respectively.
(PDF)

S1 Table Results of the PCA analysis for social openness, agricultural input and yield in the eight SE Asian countries. Bolds are the maximum absolute variable loadings among PCA axes in each variable. Only PCA axes that explained at least 10% of data variability are shown.
(PDF)

S2 Table Results of the PCA analysis for 19 climate and 12 soil variables in the eight SE Asian countries. Bolds are the maximum absolute variable loadings among PCA axes in each variable. Only PCA axes that explained at least 10% of data variability are shown.
(PDF)

S3 Table Results of correlation analysis. Bolds and italics are relationships with P≤ 0.05 and 0.05< P ≤0.1, respectively.
(PDF)

S4 Table Model selection results of multiple regression analyses. Among the 256 models considered, only the top models with low to no support relative to the best model (i.e. ΔAICc < 4) and the null model are shown.
(PDF)

S1 Text Data sources and calculation procedures. (PDF)
