# Correction: Factors affecting forest area change in Southeast Asia during 1980-2010

**DOI:** 10.1371/journal.pone.0199908

**Published:** 2018-06-25

**Authors:** Nobuo Imai, Takuya Furukawa, Riyou Tsujino, Shumpei Kitamura, Takakazu Yumoto

S1 Table is a duplicate of S1 Text. Please view the correct S1 Table below.

## Supporting information

S1 Table Results of the PCA analysis for social openness, agricultural input and yield in the eight SE Asian countries. Bolds are the maximum absolute variable loadings among PCA axes in each variable. Only PCA axes that explained at least 10% of data variability are shown.
(PDF)

## References

[1] Imai N, Furukawa T, Tsujino R, Kitamura S, Yumoto T (2018) Factors affecting forest area change in Southeast Asia during 1980–2010. PLoS ONE 13(5): e0197391 https://doi.org/10.1371/journal.pone.0197391 29763452 10.1371/journal.pone.0197391 PMC5953454

