# Supplementary figures and images for: Factors affecting forest area change in Southeast Asia during 1980-2010
Source: PLoS One. 2018 May 15;13(5):e0197391. doi: 10.1371/journal.pone.0197391 (PMC5953454; doi:10.1371/journal.pone.0197391)

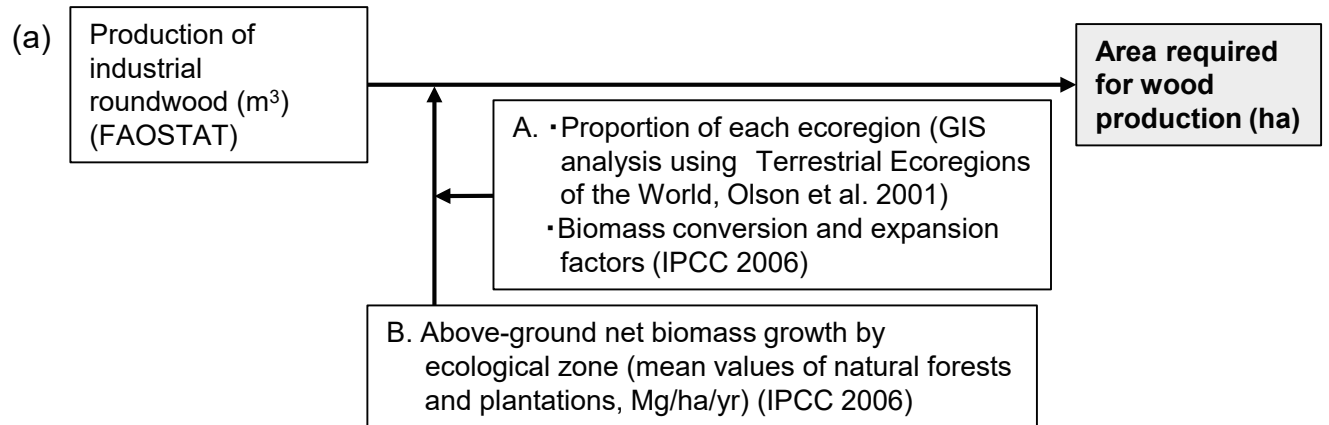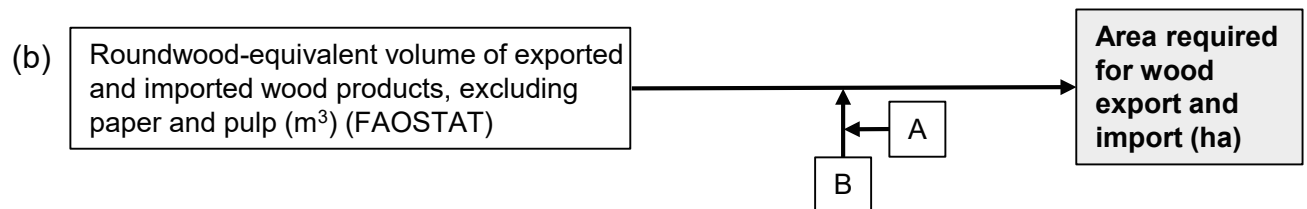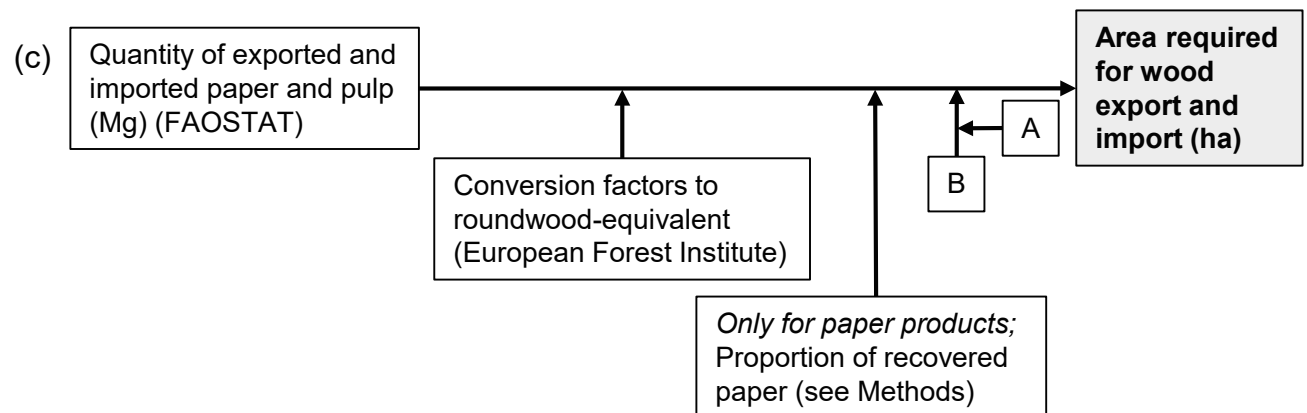

Supplement: S1 Fig — (PDF) [file pone.0197391.s001.pdf]

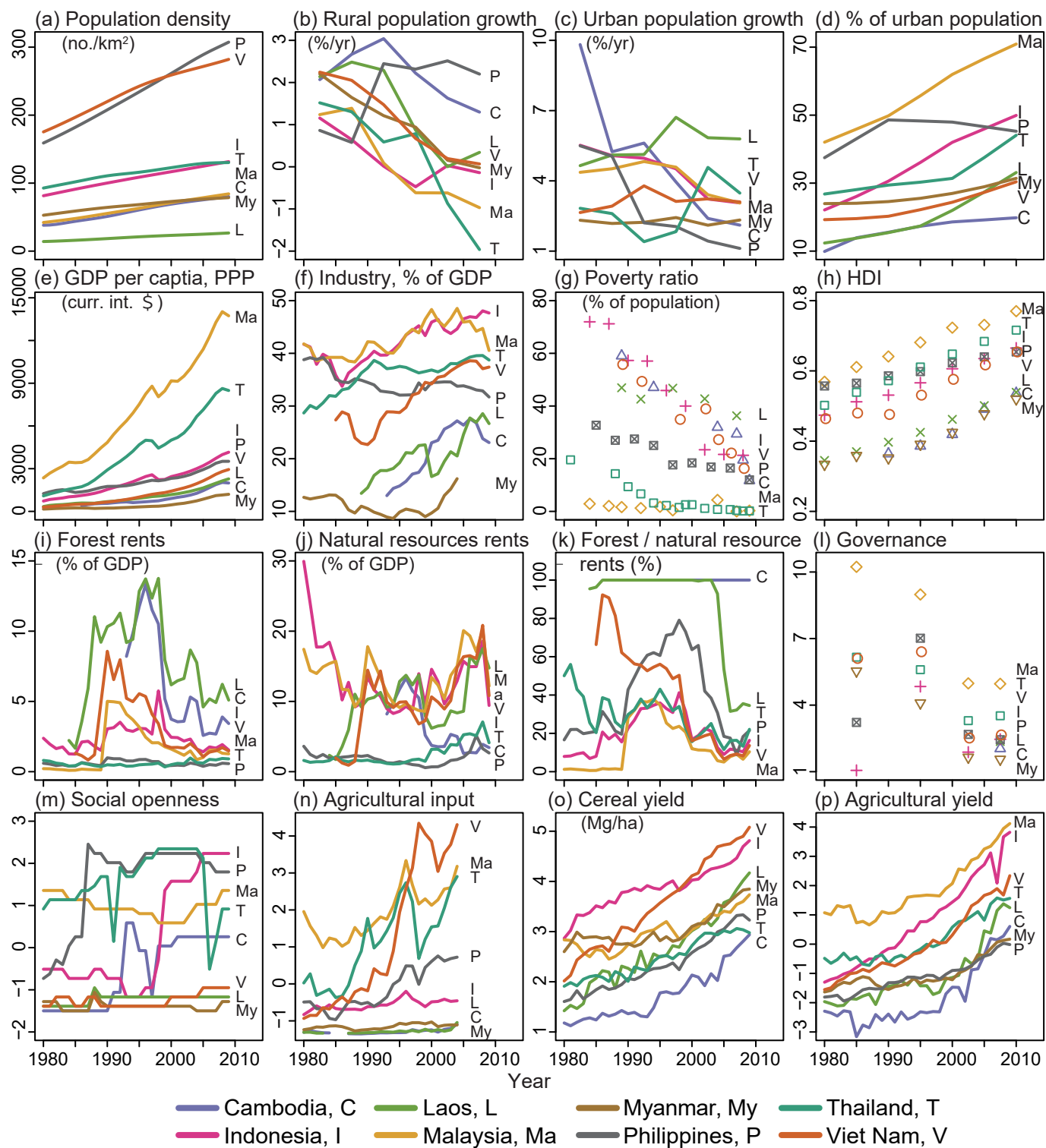

Supplement: S2 Fig — (PDF) [file pone.0197391.s002.pdf]

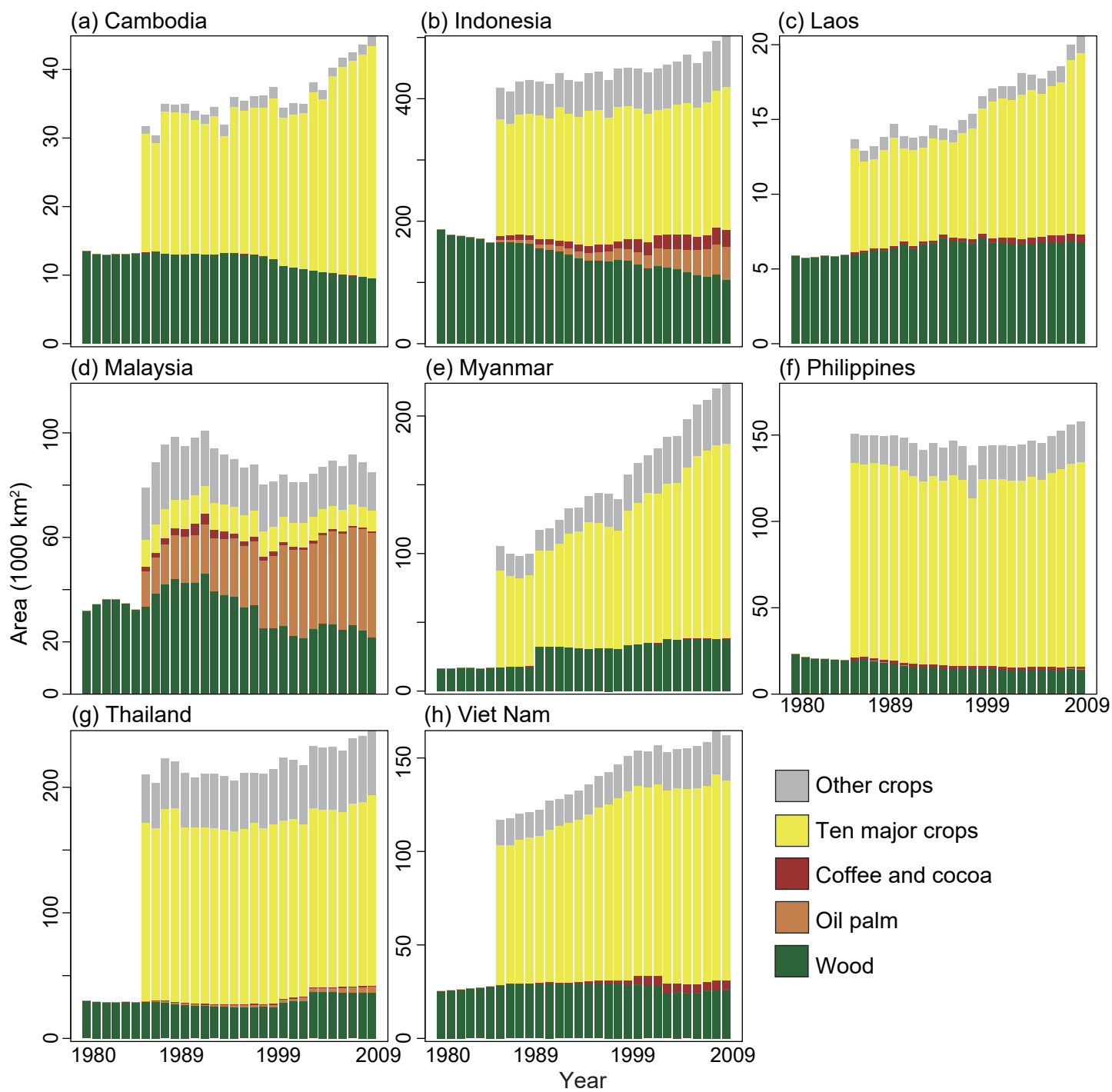

Supplement: S3 Fig — Data on food production are available only in the period 1986–2009. (PDF) [file pone.0197391.s003.pdf]

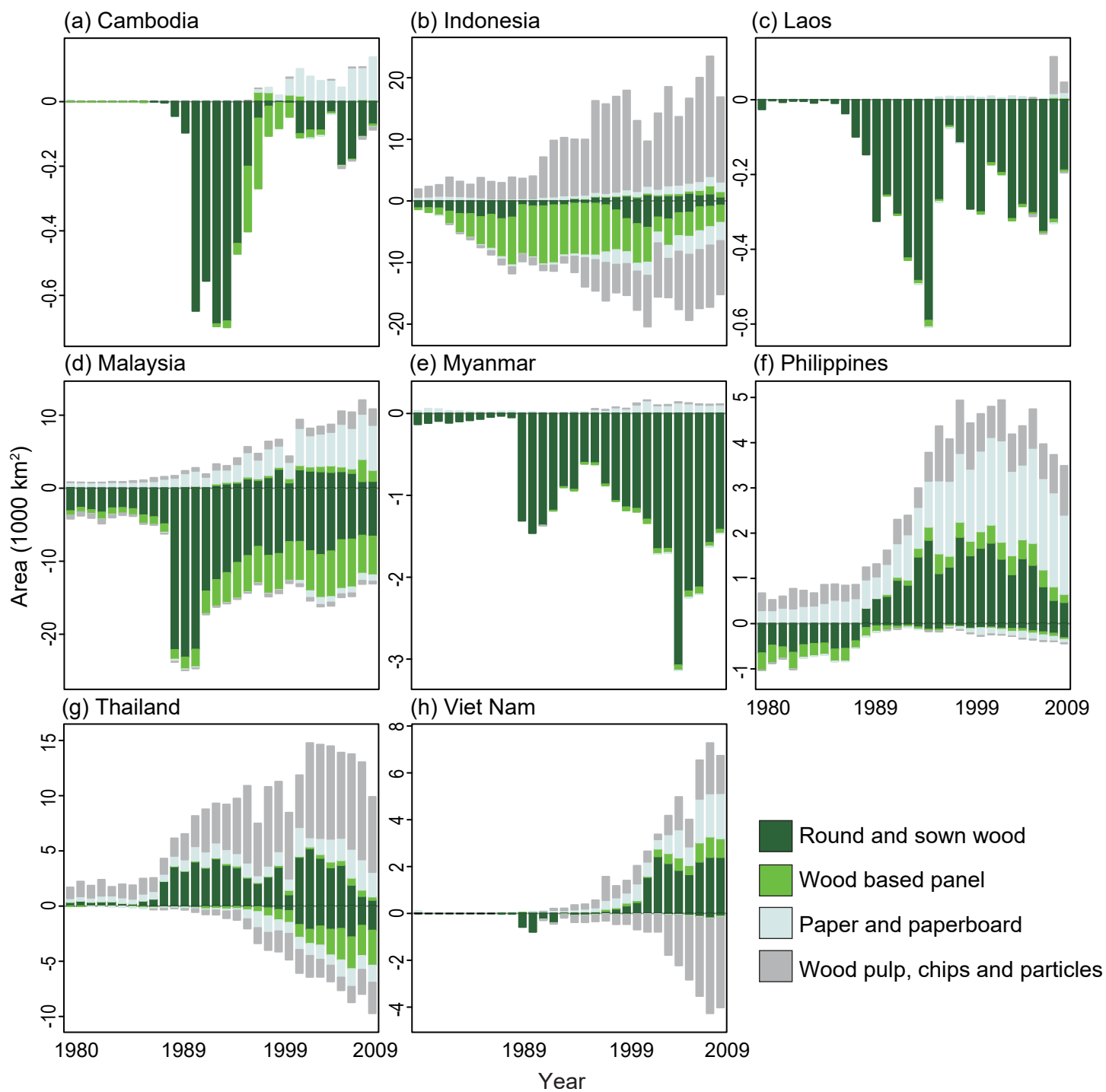

Supplement: S4 Fig — (PDF) [file pone.0197391.s004.pdf]

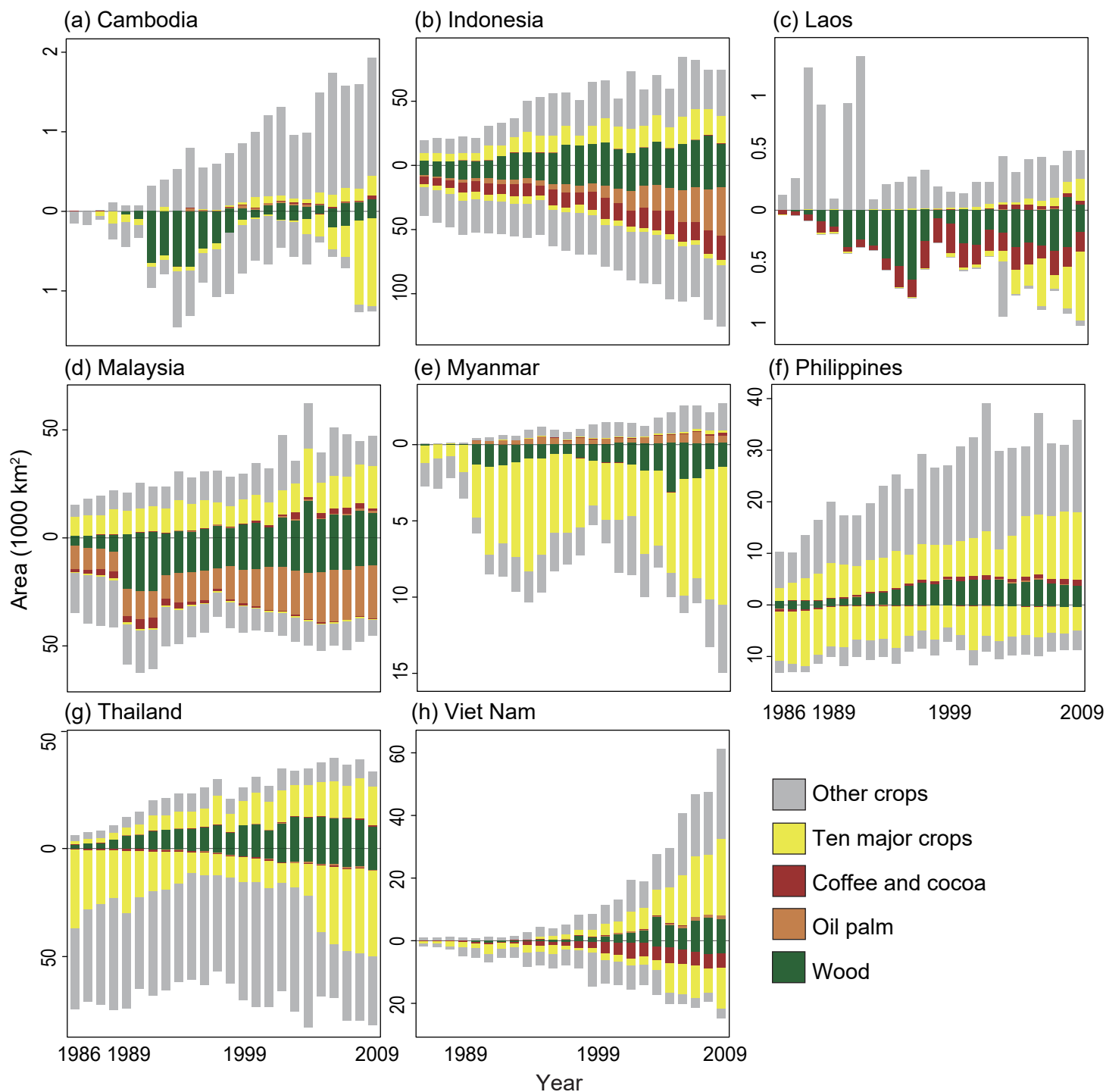

Supplement: S5 Fig — (PDF) [file pone.0197391.s005.pdf]

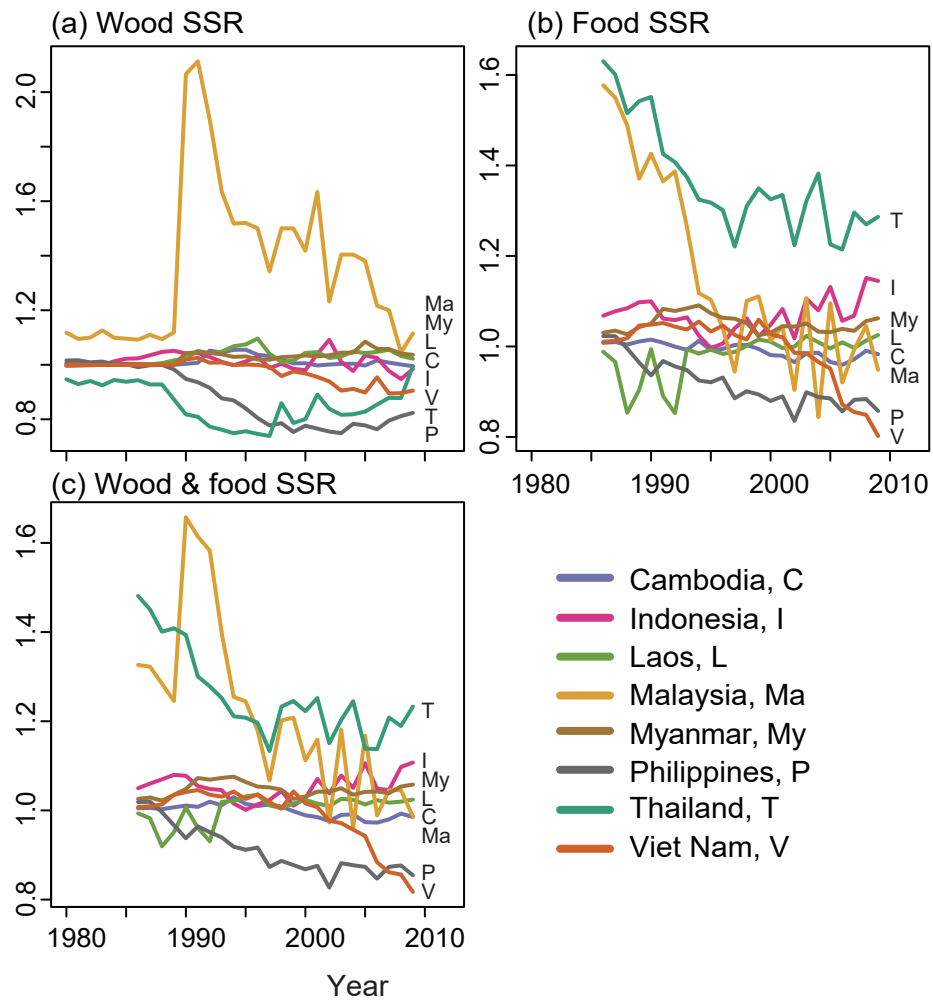

Supplement: S6 Fig — (PDF) [file pone.0197391.s006.pdf]

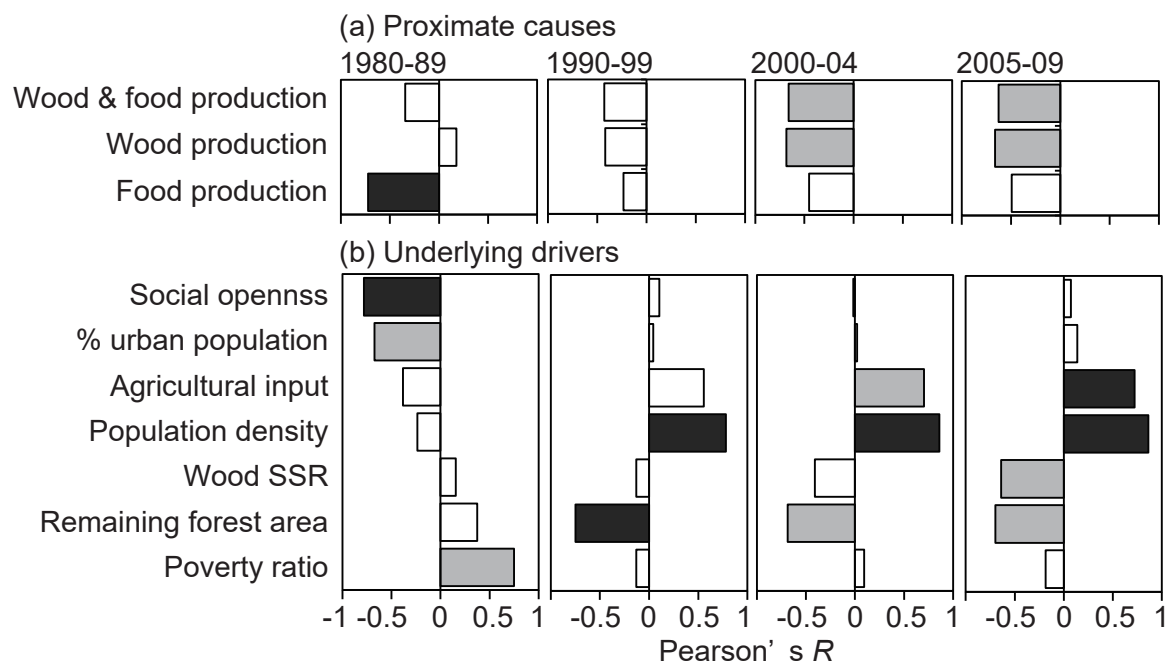

Supplement: S7 Fig — Relationships significant at P ≤0.1 in at least one of the four periods only are shown. Black, grey, and white bars are relationships with P≤ 0.05, 0.05< P ≤0.1, and P>0.1, respectively. (PDF) [file pone.0197391.s007.pdf]
