# Supplementary material for: Factors affecting forest area change in Southeast Asia during 1980-2010
Source: PLoS One. 2018 May 15;13(5):e0197391. doi: 10.1371/journal.pone.0197391 (PMC5953454; doi:10.1371/journal.pone.0197391)
Supplement: S2 Table — Bolds are the maximum absolute variable loadings among PCA axes in each variable. Only PCA axes that explained at least 10% of data variability are shown. (PDF) [file pone.0197391.s009.pdf]

	PCA1	PCA2	PCA3
Climate variables			
Standard deviation	3.30	2.54	
Proportion of variance	0.57	0.34	
Cumulative proportion	0.57	0.91	
Factor loadings			
Isothermality (BIO03)	**0.98**	0.11	
Precipitation seasonality (BIO15)	**-0.98**	-0.12	
Temperature annual range (BIO07)	**-0.98**	-0.02	
Precipitation coldest quarter (BIO19)	**0.96**	0.22	
Temperature seasonality (BIO04)	**-0.96**	0.17	
Precipitation driest quarter (BIO17)	**0.94**	0.28	
Precipitation driest month (BIO14)	**0.94**	0.29	
Minimum temperature coldest month (BIO06)	**0.93**	-0.32	
Annual precipitation (BIO12)	**0.85**	0.48	
Mean temperature driest quarter (BIO09)	**0.84**	-0.50	
Mean temperature coldest quarter (BIO11)	**0.83**	-0.52	
Diurnal temperature range (BIO02)	**-0.75**	-0.06	
Mean temperature wettest quarter (BIO08)	-0.15	**-0.98**	
Precipitation warmest quarter (BIO18)	0.05	**0.98**	
Mean temperature warmest quarter (BIO10)	-0.23	**-0.95**	
Maximum temperature warmest month (BIO05)	-0.50	**-0.81**	
Mean annual temperature (BIO01)	0.56	**-0.80**	
Precipitation wettest quarter (BIO16)	-0.29	**0.78**	
Precipitation wettest month (BIO13)	-0.36	**0.76**	
Soil variables			
Standard deviation	2.29	1.86	1.49
Proportion of variance	0.44	0.29	0.18
Cumulative proportion	0.44	0.72	0.91
Factor loadings			
Easily available water	**-0.94**	0.15	0.11
Soil moisture storage capacity (mm/m)	**-0.94**	0.15	0.11
Cation exchange capacity (soil) -Topsoil	**0.92**	-0.02	-0.08
Effective soil depth (cm)	**0.90**	0.09	0.24
Cation exchange capacity (clay) -Topsoil	**0.83**	-0.37	-0.19
Nitrogen % - Topsoil	**0.69**	-0.62	0.25
Organic carbon pool (kg/m^2/m)	-0.43	**-0.88**	-0.02
C/N ratio class - Topsoil	-0.30	**-0.79**	-0.49
pH - Topsoil	0.36	**0.78**	-0.42
Base saturation % - Topsoil	0.28	**0.70**	0.57
Soil drainage class	0.32	0.05	**-0.92**
Organic carbon % -Topsoil	0.26	-0.61	**0.66**
