# Supplementary material for: Factors affecting forest area change in Southeast Asia during 1980-2010
Source: PLoS One. 2018 May 15;13(5):e0197391. doi: 10.1371/journal.pone.0197391 (PMC5953454; doi:10.1371/journal.pone.0197391)
Supplement: S3 Table — Bolds and italics are relationships with P≤ 0.05 and 0.05< P ≤0.1, respectively. (PDF) [file pone.0197391.s010.pdf]

	% forest area in 1990		Forest-area change							
			1980-89		1990-99		2000-04		2005-09	
	P	*R*	*P*	*R*	*P*	*R*	*P*	*R*	*P*	*R*
Proximate causes										
1. Wood & food production	0.24	0.47	0.40	-0.35	0.29	-0.43	*0.07*	*-0.66*	*0.09*	*-0.63*
2. Wood production	**0.02**	**0.78**	0.68	0.18	0.30	-0.42	*0.06*	*-0.68*	*0.07*	*-0.66*
3. Food production	0.97	-0.02	**0.04**	**-0.72**	0.58	-0.23	0.26	-0.45	0.21	-0.49
4. Oil palm production	0.51	0.28	0.69	-0.17	0.89	-0.06	0.63	-0.20	0.78	-0.12
5. Stimulants production	0.44	0.32	0.74	-0.14	0.79	-0.11	0.47	0.30	0.85	0.08
6. Major crops production	0.57	-0.24	0.27	-0.45	0.74	-0.14	0.59	-0.23	0.40	-0.35
Underlying causes										
1. Population density	**0.00**	**-0.93**	0.57	-0.24	**0.02**	**0.78**	**0.01**	**0.86**	**0.01**	**0.86**
2. Total population growth	0.44	0.32	0.91	-0.05	0.84	-0.09	0.65	-0.19	0.76	-0.13
3. Rural population growth	0.32	0.40	0.17	0.54	0.62	0.21	0.86	0.08	0.87	0.07
4. Urban population growth	0.31	0.42	0.92	0.04	0.48	-0.29	0.90	-0.05	0.78	-0.12
5. Percentage of urban population	0.37	-0.37	*0.07*	*-0.67*	0.92	0.04	0.96	0.02	0.75	0.14
6. Per capita GDP	0.97	-0.02	0.19	-0.52	0.97	-0.02	0.84	-0.08	0.84	0.09
7. GDP growth	0.83	0.09	0.68	0.17	0.30	0.42	0.64	-0.20	0.41	-0.34
8. Industry	0.53	-0.29	0.40	-0.38	0.76	0.13	0.46	0.31	0.74	0.15
9. Headcount poverty ratio	0.67	0.20	*0.05*	*0.75*	0.78	-0.13	0.84	0.09	0.69	-0.19
10. Forest rents	0.31	0.51	0.27	0.54	0.68	-0.19	0.37	-0.41	0.23	-0.52
11. Total natural resources rents	0.11	0.71	0.38	0.44	0.65	-0.21	0.85	0.09	0.92	0.05
12. Proportion of forest rents	0.96	0.03	0.42	0.41	0.89	-0.07	0.28	-0.47	0.13	-0.63
13. Human development index	0.25	-0.46	0.14	-0.57	0.52	0.27	0.38	0.36	0.24	0.47
14. Governance	0.71	0.20	0.74	-0.17	0.43	0.40	0.79	-0.14	0.64	0.20
15. Social openness	0.39	-0.35	**0.02**	**-0.78**	0.80	0.11	0.97	-0.02	0.86	0.07
16. Agricultural input	0.78	-0.12	0.35	-0.38	0.15	0.56	*0.05*	*0.70*	**0.04**	**0.72**
17. Cereal yield	0.91	0.05	0.31	0.41	0.82	0.10	0.10	0.62	0.40	0.35
18. Agricultural yield	0.95	-0.03	0.68	-0.18	0.91	0.05	0.63	0.20	0.95	0.03
19. Wood SSR	0.32	0.41	0.72	0.15	0.76	-0.13	0.31	-0.41	*0.09*	*-0.64*
20. Food SSR	0.85	-0.08	0.25	-0.46	0.82	-0.09	0.78	-0.12	0.34	-0.39
21. Wood and food SSR	0.78	-0.12	0.24	-0.47	0.76	-0.13	0.50	-0.28	0.19	-0.52
22. Remaining forest area			0.36	0.37	**0.03**	**-0.75**	*0.06*	*-0.69*	*0.05*	*-0.70*
23. Median elevation	0.64	0.20	0.60	0.22	0.83	0.09	0.93	0.04	0.99	0.00
24. Total land area	0.71	0.16	0.38	0.36	0.20	-0.51	0.84	-0.09	0.55	-0.25
25. Climatic seasonality	0.96	0.02	0.61	-0.22	0.72	-0.15	0.91	-0.05	0.97	0.02
26. Soil moisture, CEC	0.59	-0.23	0.57	-0.24	0.67	-0.18	0.99	0.00	0.94	0.03
27. Lowland tropical forests	**0.05**	**-0.71**	0.17	-0.54	0.12	0.59	0.32	0.41	0.18	0.53
