# Supplementary material for: Factors affecting forest area change in Southeast Asia during 1980-2010
Source: PLoS One. 2018 May 15;13(5):e0197391. doi: 10.1371/journal.pone.0197391 (PMC5953454; doi:10.1371/journal.pone.0197391)
Supplement: S4 Table — Among the 256 models considered, only the top models with low to no support relative to the best model (i.e. ΔAICc < 4) and the null model are shown. (PDF) [file pone.0197391.s011.pdf]

Model rank	Estimate									df	logLik	AICc	Δ AICc	AICc weight
	Intercept	AGI	FP	SOP^2	SOP	URB	WSSR	POP	WP					
1 / 256	0.414	0.455	-0.008	0.395	-0.357					6	-27.92	71.19	0	0.31
2 / 256	0.954	0.494	-0.008	0.373	-0.256	-0.015				7	-26.96	72.58	1.39	0.15
3 / 256	1.717	0.492	-0.009	0.275		-0.029				6	-29.12	73.61	2.41	0.09
4 / 256	0.136	0.424	-0.007	0.373	-0.363			0.001		7	-27.65	73.97	2.78	0.08
5 / 256	0.849	0.459	-0.008	0.371	-0.354		-0.410			7	-27.75	74.16	2.97	0.07
6 / 256	0.450	0.451	-0.008	0.387	-0.359				-0.001	7	-27.87	74.41	3.22	0.06
227 / 256	-0.508									2	-49.62	103.66	32.46	0.00
IOV		1.00	1.00	0.99	0.8	0.43	0.19	0.18	0.15					
