# Supplementary material for: Factors affecting forest area change in Southeast Asia during 1980-2010
Source: PLoS One. 2018 May 15;13(5):e0197391. doi: 10.1371/journal.pone.0197391 (PMC5953454; doi:10.1371/journal.pone.0197391)
Supplement: S1 Text — (PDF) [file pone.0197391.s012.pdf]

Forest area

Data on remaining forest area (%) and the rate of change in forest area (% yr^{-1}) for the eight SE Asian countries (i.e., Cambodia, Indonesia, Laos, Malaysia, Myanmar, the Philippines, Thailand and Viet Nam) for four periods (i.e., 1980-1989, 1990-1999, 2000-2004 and 2005-2009) were obtained from the Global Forest Resources Assessment (FRA). Data on the 1980s and on the 1990s onward were obtained from FRA 1990[1] and FRA 2010[2], respectively.

We divided the study period unevenly (i.e., 1980-1989, 1990-1999, but then 2000-2004, and 2005-2009) in order to balance data consistency, data accuracy and coverage of time period. FRA2010[2], reported most accurate and consistent data on forest area among FRAs before 2010, with statistics for four time points; 1990, 2000, 2005 and 2010. However, because the trend in forest area had already started to change around 1990, especially in the three FT countries (Fig. 1), and the region was experiencing a major socioeconomic shift (S2 Fig), we extracted forest area data for 1980 from FRA 1990 to extend the coverage of time period. Forest area in 1995 is available in FRA 1995[3], but to avoid the risk of increasing inconsistency among forest area statistics, we only combined data from FRA 2010 and FRA 1990. Using the four time periods rather than the evenly divided three time periods will increase the degrees of freedom of our statistical analysis. Additionally, the effect of unevenness of time periods was minimized by using the rate of change in forest area (% yr^{-1}) when analyzing different time periods in the same statistical model.

Proximate causes

Per capita area required for wood and food production (km^2 $person^{-1}$ yr^{-1}) was used as the proximate causes of forest area change. Data on the production of industrial roundwood in roundwood-equivalent volume (m^3) was obtained from Forestry Production and Trade, FAOSTAT (http://faostat.fao.org/) for the eight countries during 1980-2009 (S1 Fig a). The annual forest area (ha yr^{-1}) required to replenish the volume of wood harvested in a given year in each country was calculated based on aboveground biomass growth (AGBG, Mg^{-1} ha^{-1} yr^{-1}) and biomass conversion and expansion factors (BCEF, Mg m^{-3})[4]. We used BCEF to convert AGBG in megagrams to cubic meters of roundwood equivalent volume, and to take into account the reduction rate of wood volume during wood extraction and processing. The AGBG varies with climatic zone (i.e., boreal, temperate, sub-tropical, and humid tropical) and forest type (i.e., natural forests and plantations). The BCEF also varies with

climatic zone, forest type and growing stock level (m^3 ha^{-1}). To estimate the share of roundwood produced from each climatic zone, we calculated the percentage land area of each climate in each country based on the World Wildlife Fund's map of terrestrial ecoregions of the world (http://www.worldwildlife.org/publications/terrestrial-ecoregions-of-the-world[5] by using ArcGIS 10.1 (ESRI). We used mean value of the AGBG between natural forests and plantations as the AGBG in each climatic zone[6]. Growing stock density (m^3 ha^{-1}) in each climatic zone and forest type was calculated based on aboveground biomass (Mg ha^{-1}) in corresponding climatic zone and forest type[4] and mean wood densities (g cm^{-3}) across regions of the world[7]. We assumed that each country's AGBG was constant throughout the study period as temporal change in AGBG is not available.

Data on annual cropland area (ha yr^{-1}) required for food production were obtained from Kastner et al. (2014)[8] who report cropland areas required to produce almost 450 crop and livestock products in over 200 nations during 1986-2009. Because expansion of lands for oil palm[9], cereal[10] and coffee production[11] is one of the principal causes of tropical deforestation in SE Asia at the local scale, we separately calculated the related cropland areas for oil palm, stimulants (coffee and cocoa), ten major crops, and the total including other crops. The ten major crops, in terms of harvested area in 2009 in the eight countries aggregated, include rice (paddy), maize, coconuts, beans (dry), cassava, sugar cane, groundnuts (with shell), sesame seed, vegetables (fresh nes) and soybeans.

Underlying causes

We collected data on demographic, economic, social, and environmental factors, as well as land-use efficiency, and wood and food trade as underlying driving forces. Demographic factors include population density (person km^{-2}), rural, urban and total annual population growth (% yr^{-1}), and percentage of urban population (S2 Fig a-d). Data was obtained from World population prospects and World urbanization prospects (http://esa.un.org/unpd/wup/), UN population division. The population data was also used to calculate per capita area required wood and food production (km^2 $person^{-1}$ yr^{-1}).

Economic factors include GDP per capita (PPP adjusted, current international USD), annual GDP growth (% yr^{-1}), industry as a percentage of GDP, headcount poverty ratio at 1.9 USD per day (% of population), forest rents (% of GDP), total natural resources rents (% of GDP), proportion of forest rents to total natural resources rents (%), and the Human

Development Index (HDI, unitless) (S2 Fig e-k). Data on GDP per capita and GDP growth rate were obtained from World economic outlook database (http://www.imf.org/external/pubs/ft/weo/2014/01/weodata/index.aspx), IMF. Data on industry, headcount poverty ratio, forest and total natural resources rents were obtained from World Bank open data (http://data.worldbank.org/). The "rents" of natural resources are the difference between the value of natural resource production at world prices and their total costs of production, representing the productiveness of the sector. Total natural resources rents are the sum of oil, gas, coal, mineral (tin, gold, lead, zinc, iron, copper, nickel, silver, bauxite and phosphate), and forest rents. HDI is a summary measure of average achievement in key dimensions of human development (health, education and income). Data on the HDI was obtained from Human development reports (http://hdr.undp.org/), UNDP. Data on industry and the HDI for Cambodia in the 1980s, natural resources rents for Cambodia in the 1980s and for Myanmar in all the study period, headcount poverty ratio for Cambodia, Laos and Viet Nam in the 1980s and for Myanmar in all the study period were not available. The values of the HDI and headcount poverty ratio between the reported years were linearly extrapolated to account for temporal changes over the study period.

Social factors include corruption and social openness (S2 Fig l,m). The Corruption Perception Index (CPI) provided by Transparency International (http://www.transparency.org/research/cpi) was used to represent corruption. The CPI scores are available for limited periods depending on the country, so the mean CPI values in 2003-2004 were used as the value corresponding to 2000-2004, except for Cambodia and Laos where the statistics are available after 2005. For time periods prior to 2000, we used data from the International Country Risk Guide (ICRG), PRS (http://epub.prsgroup.com/products/icrg/) to estimate CPI scores as in Smith et al. (2003)[12]. We obtained data on "corruption", "bureaucratic quality", and "law and order" scores from the ICRG between 1984 to present, except for Cambodia and Laos. We then produced a multiple regression model on the relationship between CPI scores and the ICRG data in 2003 in the six countries (R=0.90, P=0.013, N=6), and used this model to estimate CPI scores prior to 2000.

The index of social openness (unitless) was defined as the first axis score of principal component analysis (PCA) among the indices of polity and freedom. The "polity" score was obtained from Polity IV regime authority characteristics and transitions datasets,

INSCR (http://www.systemicpeace.org/inscrdata.html), CSP, and its score ranges from +10 (strongly democratic) to -10 (strongly autocratic). Polity scores for 1986 for Philippines and during 1981-1992 for Cambodia were not reported in the datasets. The lowest polity score in the eight countries during the last three decades (i.e., -8) was used as the substitute for the value for Cambodia in the 1980s, assuming that the civil war during the corresponding period caused severe political confusion[13]. The "political rights" and "civil liberties" scores from Freedom in the world, Freedom House (https://freedomhouse.org/) were used as indices of freedom. Both scores range from 1 (free) to 7 (not free). As in Bhattarai and Hammig (2001)[14], we aggregated the two scores to obtain an index of freedom which value varies from 2 to 14. The first axis of PCA among the two variables (polity and freedom) explained 95.9% of the total variation (S1 Table).

Land-use efficiency includes the index of agricultural input (unitless), cereal yield (Hg ha^{-1}), and the index of agricultural yield (unitless) (S2 Fig n-p). The index of agricultural input was defined as the first axis of PCA among agricultural machines import (1000 USD), pesticides import (1000 USD) and fertilizers consumption (Mg) per unit agricultural area (1000 ha). Because data on agricultural machines import after 2005 and fertilizers consumption after 2003 were not available, values between the reported years were linearly extrapolated to account for temporal changes over the study period. The PCA 1 between the three variables explained 71.0% of the total variation (S1 Table). Irrigation can also increase agricultural yield, but because irrigated land as a percentage of cropland may vary with topography and climate in a given country, we did not use any data on irrigation in the analysis. Agricultural yield was defined as the first axis of PCA among yield values of the following six crops aggregated; cereals, coarse grain, roots and tubers, vegetables and melons, fruit excluding melons, and oilcrops primary. The first axis of PCA among the six variables explained 49.6% of the total variation (S1 Table). All the agricultural data were obtained from FAOSTAT.

The self-sufficiency ratios (SSR, unitless) for wood, food, and wood and food aggregated were calculated to evaluate the contribution of wood and food trade (S6 Fig). The SSR is defined as:

$$SSR = production / (production + imports - exports) \times 100$$

The SSR was calculated based on area required for wood and food production (S3 Fig), and area associated with import/export of wood and food in the eight countries (S4 and S5 Figs)

(see below). Data on import/export of food were obtained from a trade flow analysis[8]. Data on the exports of wood products including industrial roundwood (coniferous, non-coniferous, non-coniferous tropical), sawn wood (coniferous, non-coniferous), wood based panels (fibre board, particle board, plywood, veneer sheets), paper and paperboard (newsprint, paper and paper board) and intermediate products (chips and particles, wood pulp) were obtained from Forestry Production and Trade, FAOSTAT for the eight countries during 1980-2009 (S1 Fig b). For wood pulp, news print, paper and paper board, the traded quantities in megagrams were converted to roundwood-equivalent volume (m^3) by using conversion factors for each product from Observatory of the European Forest Economy (https://sites.google.com/site/forestproductstrade/metadata/analysed-products), European Forest Institute (S1 Fig c). To only account for paper (news print, paper and paper board) produced from virgin fiber, we calculated the share of recovered paper in total paper production for each country, and the paper exports of each country were multiplied by the share not from recovered paper, as in Kastner et al. (2011)[15] (S1 Fig c). The area required for replenishing the volume of exported wood products was calculated based on the AGBG in each country, as mentioned earlier. Data on the imports of wood products for the eight "reporter" countries from 174 "partner" countries during 1997-2009 were obtained from Forestry Trade Flows, FAOSTAT. The AGBG, roundwood-equivalent volume of wood pulp and paper products, and the share of recovered paper were calculated for the 174 countries. Then, the area required for producing the quantity of imported wood products was calculated for the eight countries (S1 Fig b,c). The bilateral wood trade data before 1997 is not available in FAOSTAT. Data on the imports of wood products for the eight countries during 1981-1996 (without data on partner countries) were obtained from Forestry Production and Trade, FAOSTAT. For each of wood products, mean proportions of imports coming from the partner countries in the period 1997-1999 were calculated, and were assumed to remain constant before 1997.

Environmental factors that might underlie the change in forest area include remaining forest area at the beginning of each period (%), median elevation (m), total land area (km^2), climate and soil (unitless), and percentage land area of lowland tropical forests as potential natural vegetation (%). We downloaded elevation and 19 climate variables from WorldClim (http://www.worldclim.org/)[16] with a *ca.* 1-km resolution, and 12 soil variables from GeoNetwork (http://www.fao.org/geonetwork/srv/en/main.home) with a *ca.* 10-km

resolution. Median values of elevation, climate and soil variables in each of the eight countries were calculated using ArcGIS 10.1. We conducted PCA for both the climate and soil variables. The first axis of PCA for climate variables represented the climatic seasonality and explained 57.4% of the total variation, while the first axis of PCA for soil variables represented soil moisture and cation exchange capacity and explained 43.6% of the total variation (S2 Table). Lowland tropical forests have a higher risk of conversion to cropland, compared to montane forests given the warmer climate and flatter topography. Percentage land area of lowland tropical forests (including lowland wet, moist, and dry forests, and peat and freshwater swamp forests) as potential natural vegetation in each country was estimated based on terrestrial ecoregions of the world[5].

References

1. FAO. Forest resources assessment 1990: global synthesis. FAO Forestry Paper No. 124. Rome; 1995.
2. FAO. Global forest resources assessment 2010. Rome; 2010.
3. FAO. State of the world's forests. Oxford; 1997.
4. IPCC. 2006 IPCC Guidelines for National Greenhouse Gas Inventories, Prepared by the National Greenhouse Gas Inventories Programme, Eggleston HS, Buendia L, Miwa K, Ngara T, Tanabe K, editors. IGES, Japan; 2006.
5. Olson DM, Dinerstein E, Wikramanayake ED, Burgess ND, Powell GVN, Underwood EC et al. Terrestrial ecoregions of the world: a new map of life on earth. Bioscience 2001;51: 933-938.
6. D'Annunzio R, Sandker M, Finegold Y, Min Z. Projecting global forest area towards 2030. For Ecol Manage. 2015;352: 124-133.
7. Chave J, Coomes D, Jansen S, Lewis SL, Swenson NG, Zanne AE. Towards a worldwide wood economics spectrum. Ecol Lett. 2009;12: 351-366.
8. Kastner T, Erb K-H, Haberl H. Rapid growth in agricultural trade: effects on global area efficiency and the role of management. Environ Res Lett. 2014;9: 34015.
9. Carlson KM, Curran LM, Asner GP, Pittman AM, Trigg SN, Marion Adeney J. Carbon emissions from forest conversion by Kalimantan oil palm plantations. Nat Clim Chang. 2013;3: 283-287.
10. Phalan B, Bertzky M, Butchart SHM, Donald PF, Scharlemann JPW, Stattersfield AJ

et al. Crop expansion and conservation priorities in tropical countries. PLoS One 2013;8: e51759.

11. Gaveau DLA, Linkie M, Suyadi, Levang P, Leader-Williams N. Three decades of deforestation in southwest Sumatra: effects of coffee prices, law enforcement and rural poverty. Biol Conserv. 2009;142: 597-605.

12. Smith RJ, Muir RDJ, Walpole MJ, Balmford A, Leader-Williams N. Governance and the loss of biodiversity. Nature 2003;426: 67-70.

13. de Zeeuw J. “Sons of war”: parties and party systems in post-war El Salvador and Cambodia. Democratization 2010;17: 1176-1201.

14. Bhattarai M, Hammig M. Institutions and the environmental Kuznets curve for deforestation: a crosscountry analysis for Latin America, Africa and Asia. World Dev. 2001;29: 995-1010.

15. Kastner T, Erb K-H, Nonhebel S. International wood trade and forest change: a global analysis. Glob Environ Chang. 2011;21: 947-956.

16. Hijmans RJ, Cameron SE, Parra JL, Jones PG, Jarvis A. Very high resolution interpolated climate surfaces for global land areas. Int J Climatol. 2005;25: 1965-1978.
