# Supplementary material for: Correction: Factors affecting forest area change in Southeast Asia during 1980-2010
Source: PLoS One. 2018 Jun 25;13(6):e0199908. doi: 10.1371/journal.pone.0199908 (PMC6016896; doi:10.1371/journal.pone.0199908)
Supplement: S1 Table — Bolds are the maximum absolute variable loadings among PCA axes in each variable. Only PCA axes that explained at least 10% of data variability are shown. (PDF) [file pone.0199908.s001.pdf]

	PCA1	PCA2	PCA3	PCA4
Social openness				
Standard deviation	1.39			
Proportion of variance	0.96			
Cumulative proportion	0.96			
Factor loadings				
Polity	**0.71**			
Freedom	**-0.71**			
Agricultural input				
Standard deviation	1.46	0.84		
Proportion of variance	0.71	0.24		
Cumulative proportion	0.71	0.95		
Factor loadings				
Fertilizers	**0.64**	-0.28		
Pesticides	**0.63**	-0.35		
Agricultural machines	0.45	**0.89**		
Agricultural yield				
Standard deviation	1.72	1.11	0.89	0.79
Proportion of variance	0.50	0.21	0.13	0.10
Cumulative proportion	0.50	0.70	0.83	0.94
Factor loadings				
Coarse grain	**0.47**	-0.40	0.08	0.09
Vegetables and melons	0.31	**0.60**	0.53	-0.18
Roots and tubers	0.41	**-0.48**	0.20	0.36
Fruit excluding melons	0.36	0.22	**-0.82**	-0.03
Oilcrops primary	0.44	0.43	0.01	**0.48**
Cereals	0.44	-0.16	0.01	**-0.77**
